# Giant Clear Cell Renal Cell Carcinoma with Focal Chromophobe-Like Morphology in a Young Adult Presenting with Gastrointestinal Symptoms: A Case Report

**DOI:** 10.1159/cro/abzag020

**Published:** 2026-09-30

**Authors:** Salem Hakouk, Omar Al-Abed, Mohammad Alaa Aldakak, Anas Saad, Ahmad Awaid, Lina Assad, Ibrahim Barghouth

**Affiliations:** a Department of Urology, National University Hospital, Faculty of Medicine, Damascus University, Damascus, Syrian Arab Republic; b Faculty of Medicine, Damascus University, Damascus, Syrian Arab Republic

**Keywords:** Clear cell renal cell carcinoma, Giant renal tumor, Chromophobe-like morphology, Young adult, Radical nephrectomy

## Abstract

**Introduction:**

Renal cell carcinoma is increasingly detected incidentally in the modern imaging era, and presentation as a giant renal mass is uncommon. Clear cell renal cell carcinoma is the most frequent histological subtype; however, unusual morphological patterns may create diagnostic challenges, particularly in young patients.

**Case Presentation:**

A 31-year-old Syrian man presented with a 1-year history of recurrent colicky abdominal pain, postprandial dyspepsia, vomiting, and marked unintentional weight loss. Physical examination revealed a large firm left flank mass, while urinalysis showed microscopic hematuria without gross hematuria. Contrast-enhanced computed tomography demonstrated a giant left renal mass measuring approximately 18 × 17 × 23 cm, with a maximum diameter of about 24 cm on multiplanar images, heterogeneous enhancement, and central necrosis, without renal vein or inferior vena cava tumor thrombus. The patient underwent open transperitoneal radical nephrectomy with lymph node excision. Gross examination revealed a 3,200-g nephrectomy specimen containing a 23-cm tan-white mass with necrosis and hemorrhage extending to the renal sinus, capsule, and perinephric fat. Microscopy showed nests, tubules, and alveolar structures composed of polygonal cells with clear to pale eosinophilic cytoplasm and Fuhrman grade 2 nuclei. Immunohistochemistry demonstrated cytokeratin and CD10 positivity, with CK7 and CD117 negativity, supporting the diagnosis of clear cell renal cell carcinoma with focal eosinophilic/chromophobe-like morphology. Surgical margins were negative, and lymph nodes were uninvolved.

**Conclusion:**

This case highlights an unusual giant clear cell renal cell carcinoma in a young adult presenting predominantly with gastrointestinal symptoms. It emphasizes the importance of considering renal malignancy in patients with unexplained abdominal symptoms and the value of accurate pathological classification and long-term surveillance.

## Introduction

Renal cell carcinoma (RCC) is a malignant tumor arising from the renal parenchyma and represents one of the major adult urologic malignancies [1]. Clear cell renal cell carcinoma (ccRCC) is the most common histological subtype, accounting for approximately 60–75% of adult renal tumors [2]. RCC is more frequently encountered in older adults, particularly during the fifth to seventh decades of life, whereas renal tumors are relatively uncommon in children and young adults [3]. The classic triad of hematuria, flank pain, and a palpable abdominal mass is now uncommon and is usually associated with advanced disease [1]. Because ultrasonography and computed tomography are increasingly used in clinical practice, many renal tumors are detected incidentally at smaller sizes and earlier stages [3]. Consequently, presentation as a huge or giant renal mass has become unusual in the modern imaging era [4, 5]. Giant RCC has been described as a renal tumor with a volume exceeding 1,000 cm^3^, and such tumors are considered rare because of the generally slow growth rate of RCC and improvements in diagnostic imaging [5]. Large renal tumors may produce nonspecific or compressive symptoms, including abdominal pain, early satiety, anorexia, weakness, weight loss, or other atypical presentations rather than urinary symptoms [6]. Diagnosis is primarily based on imaging modalities such as contrast-enhanced computed tomography, whereas definitive classification requires histopathological examination and, when necessary, immunohistochemical evaluation [2]. Surgical resection remains the mainstay of treatment for localized RCC, while giant or advanced tumors may require multidisciplinary planning and multimodal management [1, 5]. In young adults, RCC should also raise consideration of hereditary syndromes, including von Hippel-Lindau disease, Birt-Hogg-Dubé syndrome, tuberous sclerosis, and hereditary papillary RCC, particularly when tumors are bilateral, multifocal, or associated with syndromic manifestations [3, 7]. Here, we present a case of a giant ccRCC with focal eosinophilic/chromophobe-like morphology in a 31-year-old Syrian man presenting predominantly with gastrointestinal symptoms and profound weight loss. The work has been reported in line with the CARE checklist criteria [8].

## Case Presentation

A 31-year-old Syrian male presented with a 1-year history of recurrent colicky abdominal pain, postprandial dyspepsia, vomiting, and marked unintentional weight loss from 112 kg to 86 kg, corresponding to a decrease in body mass index from 31.3 to 24 kg/m^2^. He had no significant past medical history and no family history of gastrointestinal or renal tumors. He reported a smoking history of approximately 54 pack-years. Because the patient initially lived in a rural area and his symptoms were predominantly gastrointestinal, he received treatment for presumed *Helicobacter pylori* infection without clinical improvement.

During subsequent clinical evaluation, physical examination revealed a large, firm, palpable mass in the left flank that moved with respiration. The patient denied gross hematuria and had no symptoms suggestive of paraneoplastic syndromes. Laboratory investigations on admission showed hemoglobin of 13 g/dL, white blood cell count of 9.3 × 10^9^/L with 71% neutrophils, platelet count of 300 × 10^9^/L, serum sodium of 143 mEq/L, potassium of 4.5 mEq/L, creatinine of 1.1 mg/dL, prothrombin time of 85%, and serum glucose of 90 mg/dL. Urinalysis revealed 50 red blood cells per high-power field, consistent with microscopic hematuria.

Contrast-enhanced computed tomography of the abdomen demonstrated a large, well-defined left renal mass with smooth margins, measuring approximately 18 × 17 × 23 cm, with a maximum measured diameter of approximately 24 cm on multiplanar images. The mass compressed and expanded the remaining calyceal system and showed heterogeneous enhancement with areas of central necrosis. It extended beyond the renal contour and was associated with surrounding nodular lesions, the largest of which was located near the splenorenal region and measured approximately 1.8 cm in diameter. The left renal artery and vein were patent, with no evidence of renal vein invasion or tumor thrombus. The contralateral kidney, liver, spleen, pancreas, adrenal glands, urinary bladder, prostate, and major retroperitoneal vessels were unremarkable on imaging. The radiological differential diagnosis included clear cell sarcoma, Wilms tumor, and renal oncocytoma, and histopathological evaluation was recommended (Fig. 1).

**Fig. 1.**
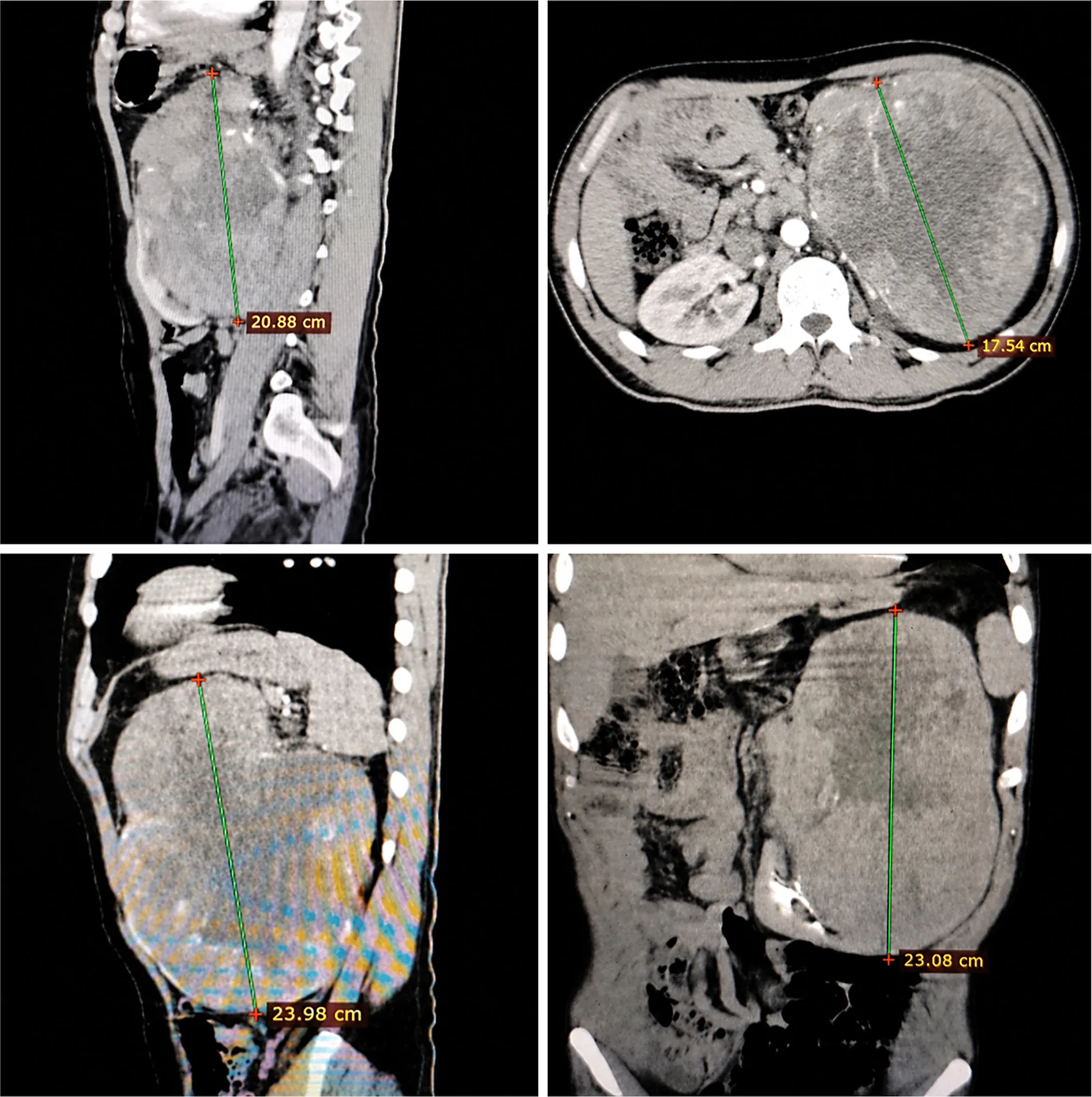
Preoperative contrast-enhanced computed tomography findings. Multiplanar contrast-enhanced abdominal computed tomography demonstrated a giant, well-defined left renal mass with heterogeneous enhancement and internal areas of low attenuation suggestive of necrosis. The mass occupied most of the left abdomen and displaced adjacent structures. The maximum tumor diameter measured approximately 24 cm on sagittal reconstruction. Panels are arranged from left to right and top to bottom: sagittal, axial, sagittal, and coronal reconstructed views.

The case was reviewed by a multidisciplinary medical team. Given the large size of the tumor, its renal origin, and the absence of radiological evidence of renal vein or inferior vena cava invasion, primary surgical management was selected. The patient underwent open transperitoneal radical nephrectomy through a midline laparotomy on January 21, 2026. After entering the peritoneal cavity, the left colon was mobilized medially to expose the retroperitoneal space. A giant left renal mass occupying the left flank and displacing adjacent structures was identified. Dissection proceeded toward the renal hilum, where early vascular control was achieved by ligation of the renal artery followed by the renal vein. The kidney was carefully dissected en bloc with the perinephric fat, Gerota’s fascia, and ureteral segment. Significant adhesions to adjacent structures, including the spleen, pancreas, psoas muscle, and diaphragm, were carefully released to achieve complete oncological resection. An enlarged lymph node adjacent to the renal artery was identified and excised for pathological evaluation (Fig. 2).

**Fig. 2.**
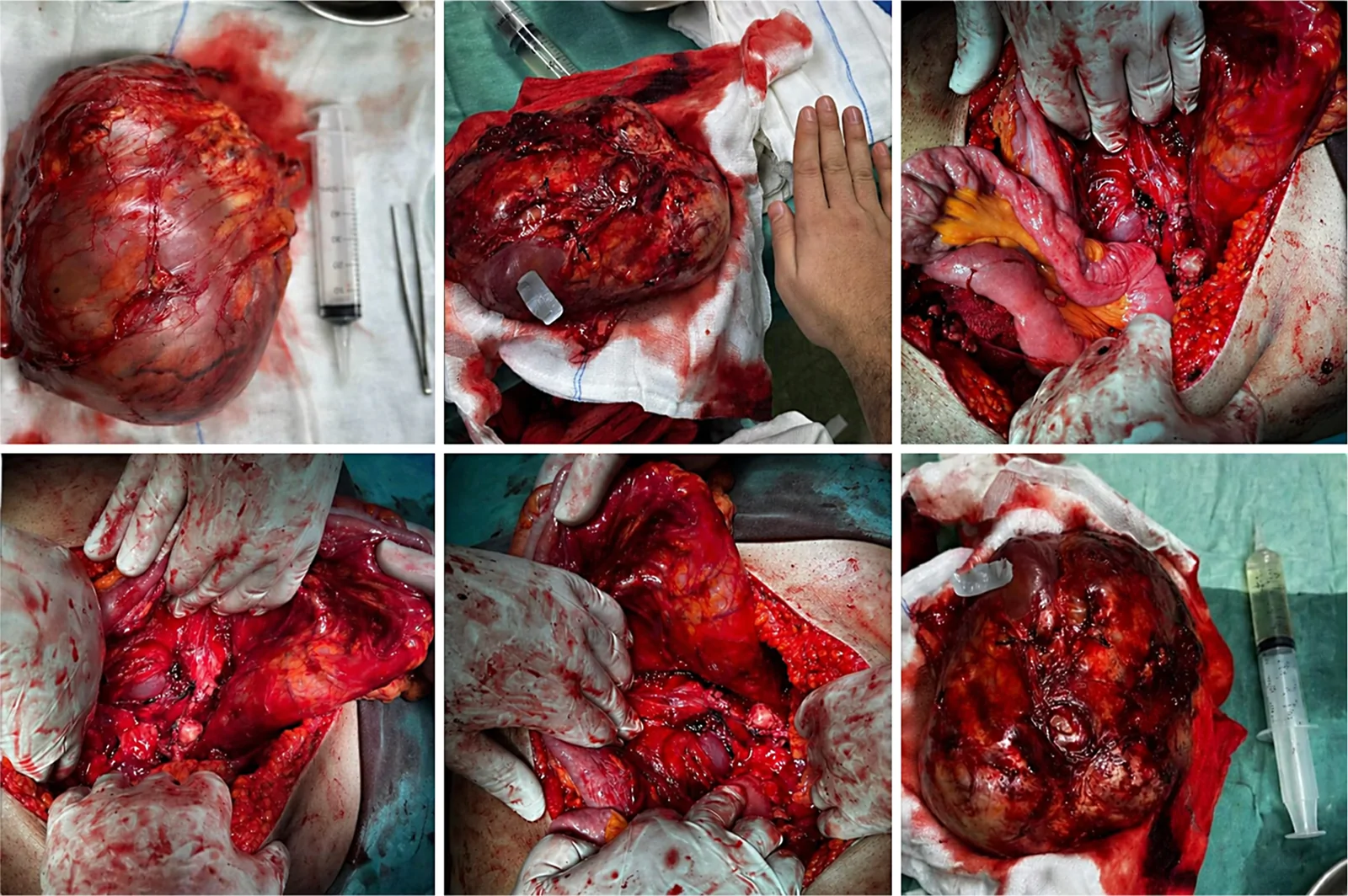
Intraoperative and gross surgical findings. Intraoperative photographs obtained during open transperitoneal radical nephrectomy demonstrate a large left renal mass occupying the operative field, with careful mobilization and dissection from adjacent structures. Gross photographs of the resected specimen show a large, well-circumscribed, congested, and vascular renal mass, with adjacent surgical instruments and the surgeon’s hand used for scale. Panels are arranged from left to right and top to bottom.

The postoperative course was uneventful. The patient remained hospitalized for 3 days; the drain was removed on the fourth postoperative day, and he was discharged in good general condition.

Gross pathological examination revealed a total nephrectomy specimen with surrounding perinephric fat measuring 23 × 16 × 13.5 cm, with an attached ureter measuring 4 cm in length. The specimen weighed 3,200 g. On sectioning, a firm dark tan-white mass measuring 23 cm in greatest dimension was identified. The tumor demonstrated geographic necrosis and hemorrhage and extended to the renal sinus, renal capsule, and perinephric fat.

Microscopic examination of hematoxylin and eosin-stained sections demonstrated a renal epithelial neoplasm arranged predominantly in nests, tubules, and alveolar structures separated by delicate fibrovascular septa. The tumor cells were large and polygonal, with clear to pale eosinophilic or flocculent cytoplasm, distinct cytoplasmic borders, and mildly enlarged irregular nuclei with small discernible nucleoli, corresponding to Fuhrman nuclear grade 2. Some areas showed classic clear cell morphology, whereas other areas demonstrated eosinophilic cytoplasm, prominent cell borders, and perinuclear clearing, raising a chromophobe-like morphological differential. Immunohistochemistry showed strong expression of cytokeratin and CD10, supporting ccRCC differentiation. CK7 and CD117 immunostaining were negative (not shown), arguing against a definitive chromophobe RCC component (Fig. 3).

**Fig. 3.**
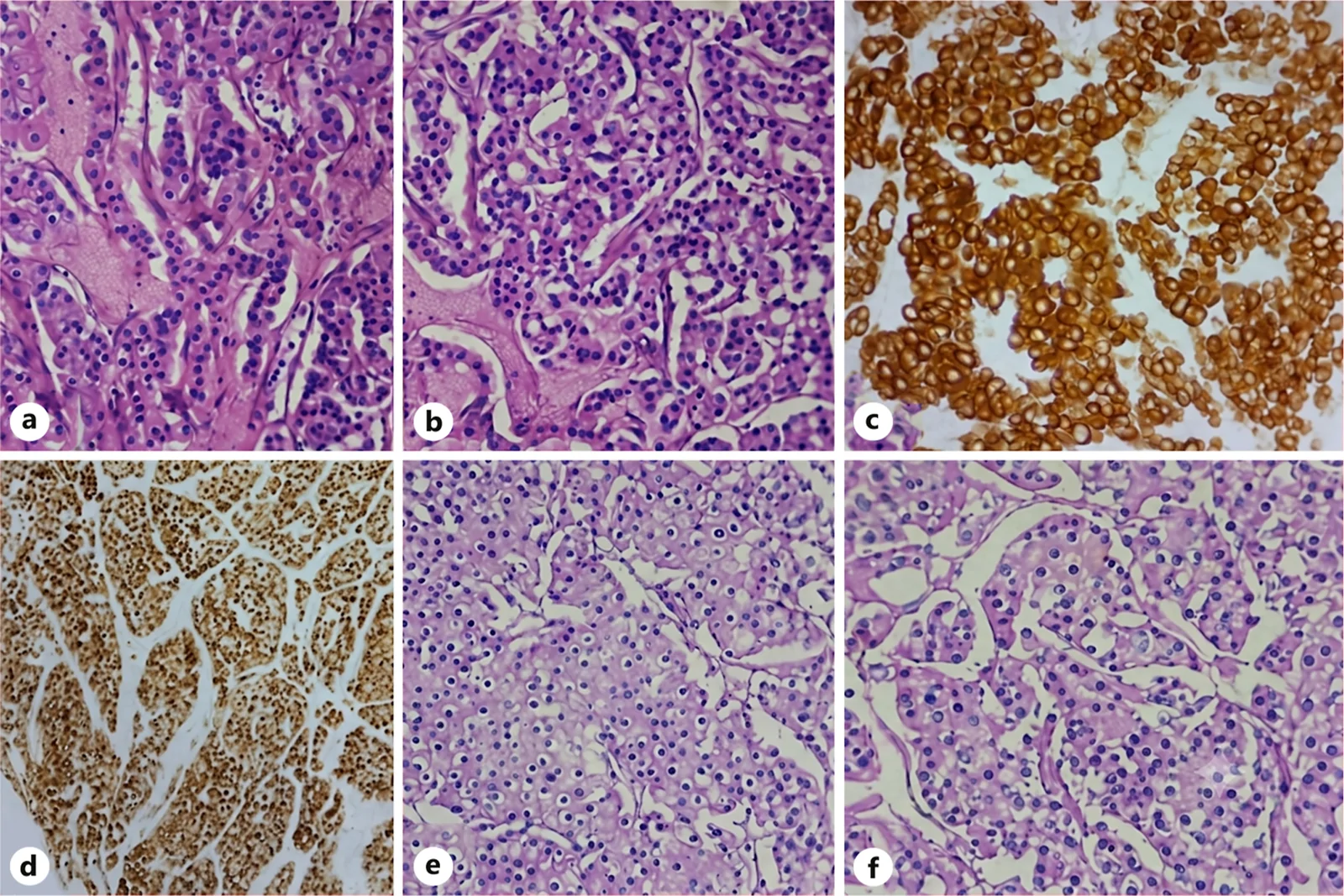
Histopathological and immunohistochemical findings. **a**, **b**, **e**, **f** Hematoxylin and eosin (H&E)-stained sections demonstrating a renal epithelial neoplasm arranged in nests, tubules, and alveolar structures separated by delicate fibrovascular septa. Tumor cells show clear to pale eosinophilic/flocculent cytoplasm, distinct cytoplasmic borders, and mildly enlarged irregular nuclei with small discernible nucleoli, corresponding to Fuhrman nuclear grade 2. Some areas demonstrate classic clear cell morphology, whereas other areas show eosinophilic cytoplasm, prominent cell borders, and perinuclear clearing, resulting in a focal chromophobe-like morphological appearance. **c** Cytokeratin (CK) immunohistochemical staining demonstrating diffuse strong positivity, supporting renal epithelial differentiation. **d** CD10 immunohistochemical staining demonstrating strong positivity, supporting ccRCC differentiation.

Based on the overall morphological and immunohistochemical profile, the tumor was classified as ccRCC with focal eosinophilic/chromophobe-like morphology, Fuhrman nuclear grade 2. No renal vein tumor thrombus was identified. The ureteral and vascular surgical margins were free of tumor. The excised lymph nodes showed no evidence of metastatic carcinoma, with 0 of 2 lymph nodes involved.

At 6-month follow-up, the patient reported complete resolution of his gastrointestinal symptoms and significant weight regain, reaching 104 kg with a body mass index of 29.1 kg/m^2^. Serum creatinine remained within the normal range, and follow-up abdominal computed tomography showed no evidence of local recurrence or metastatic disease. Written informed consent was obtained from the patient for publication of this case report and accompanying images.

## Discussion

This case is notable for the combination of young age, giant tumor size, predominantly gastrointestinal presentation, and an unusual focal eosinophilic/chromophobe-like morphology within an otherwise ccRCC. Although RCC classically presents with flank pain, gross hematuria, and a palpable abdominal mass, this triad is usually associated with advanced disease and is not present in many contemporary cases [1]. Our patient initially presented with colicky abdominal pain, postprandial dyspepsia, vomiting, and marked weight loss, without gross hematuria, which delayed recognition of a renal origin and led initially to treatment for presumed *H. pylori* infection. A similar diagnostic challenge has been reported in a 42-year-old patient with giant RCC presenting as chronic low back pain, where diagnosis was difficult without appropriate imaging modalities [9]. Compressive gastrointestinal symptoms have also been described in giant renal tumors, including a 59-year-old woman with giant chromophobe RCC who presented with abdominal pain, early satiety, anorexia, weakness, and weight loss [6]. These comparisons support the idea that giant renal tumors may present with nonspecific or extra-urinary symptoms, especially when their growth is slow and progressive.

The size of the tumor in our case is another important feature, as imaging showed a mass measuring approximately 18 × 17 × 23 cm, with a maximum diameter of about 24 cm on multiplanar images, and the nephrectomy specimen weighed 3,200 g. This size is comparable to previously reported giant ccRCC cases, including a 48-year-old man with a 2,695-g ccRCC measuring 22 × 17 × 14 cm associated with renal vein thrombus, liver and lung metastases, and death 6 months after surgery [1]. Another reported giant ccRCC measured 22 × 25 × 18 cm and weighed 5 kg, with Fuhrman grade 2 histology and no complaints during 1 year of follow-up despite positive surgical margins [5]. A separate case of giant RCC presenting as low back pain had a final specimen measuring 23.5 × 18.0 × 8.5 cm and showed renal sinus fat and vena cava wall invasion after neoadjuvant nivolumab and cabozantinib [10]. Compared with these cases, our patient had a similarly large tumor but no renal vein tumor thrombus, negative vascular and ureteral margins, and no nodal metastasis in the two excised lymph nodes. However, our case should not be considered biologically indolent solely because of the absence of venous thrombus or nodal disease, since the tumor extended to the renal sinus, renal capsule, and perinephric fat, supporting an advanced local pathological stage. The case of the world’s largest reported RCC, measuring 43 cm and weighing 13 kg, also illustrates that tumor size alone does not fully determine pathological stage, as that tumor was a low-grade type 1 papillary RCC confined to the kidney and staged as pT2b [4]. Therefore, our case reinforces that local invasion, vascular involvement, histologic grade, subtype, and metastatic status should be interpreted together rather than relying on size alone.

The histopathological interpretation in our case also deserves careful discussion because the tumor showed classic clear cell areas as well as focal eosinophilic/chromophobe-like morphology. Classic ccRCC is usually composed of nests of cells with optically clear cytoplasm and an intricate branching fibrovascular network, but ccRCC can show other morphologic patterns including eosinophilic cytoplasm, pseudopapillary architecture, and other diagnostically challenging appearances [2]. In our case, the tumor showed nests, tubules, and alveolar structures with clear to pale eosinophilic/flocculent cytoplasm, distinct cell borders, and Fuhrman nuclear grade 2 nuclei. The immunohistochemical profile showed strong cytokeratin and CD10 expression, supporting ccRCC differentiation, while CK7 and CD117 negativity argued against a definitive chromophobe RCC component. This interpretation is supported by the chromophobe RCC literature, in which CK7 and CD117 positivity are described as characteristic findings of chromophobe RCC [6]. Because renal tumors with unusual morphology can create broad diagnostic differentials, careful integration of morphology and immunohistochemistry is essential for accurate classification [11]. Similar diagnostic challenges have been described in rare combined ccRCC and chromophobe RCC cases, where distinguishing overlapping morphological features requires careful correlation between histopathology and immunohistochemical findings [12]. Accordingly, the most cautious diagnostic wording for our case is ccRCC with focal eosinophilic/chromophobe-like morphology, rather than definite mixed clear cell and chromophobe RCC.

The young age of our patient is another clinically relevant point, as renal tumors most often occur in older adults and are relatively uncommon in young adults [3]. In a large series of renal tumors in patients younger than 45 years, RCC remained the most common malignant tumor, and ccRCC was the most frequent subtype among renal tumors in this age group [3]. Nevertheless, younger patients are more likely than older adults to have hereditary or syndromic associations, including von Hippel-Lindau disease, Birt-Hogg-Dubé syndrome, tuberous sclerosis, and hereditary papillary RCC [3]. A recent case of synchronous bilateral RCC as the initial presentation of von Hippel-Lindau disease emphasized that young patients may present without family history and still require syndromic suspicion and multidisciplinary evaluation [7]. Our patient had no reported family history and no bilateral or multifocal renal disease; however, his young age at diagnosis still raises consideration of hereditary RCC syndromes. Genetic counseling may be considered in young patients with RCC even in the absence of a positive family history, as identifying hereditary predisposition may influence surveillance strategies and family counseling. Genetic testing was not performed in our case, which should be acknowledged as a limitation, particularly because hereditary RCC syndromes may have implications for long-term management [3, 7].

Surgical management was appropriate in our case because the tumor was very large, localized on imaging, and had no radiological renal vein or inferior vena cava tumor thrombus. Surgical resection is described as the primary treatment for localized RCC and an important procedure for achieving long-term survival [1]. For giant renal tumors, open surgery may be required because these masses occupy substantial abdominal space and can have complex relationships with adjacent organs and vessels [6]. Our patient underwent open transperitoneal radical nephrectomy with excision of an enlarged lymph node. The procedure required careful mobilization of the left colon, early renal hilar control, and en bloc removal of the kidney with surrounding perinephric tissues and ureteral segment. The postoperative course was uneventful, and the patient was discharged after drain removal on postoperative day 4.

At 6-month follow-up, his gastrointestinal symptoms had completely resolved, weight had increased from 86 kg to 104 kg, serum creatinine remained normal, and abdominal CT showed no evidence of local recurrence or metastatic disease. This favorable early outcome contrasts with reported giant ccRCC cases complicated by venous thrombus, metastasis, sarcomatoid or rhabdoid differentiation, or poor short-term survival [1, 9, 12]. However, the relatively short follow-up period remains an important limitation of this report. Although no recurrence was detected during the first 6 months after surgery, long-term surveillance remains essential because large tumor size, pT3a local invasion, and unusual morphology may still carry a risk of delayed recurrence beyond the early follow-up period [1, 11].

The clinical significance of this case lies in several aspects. First, it demonstrates that giant RCC may present predominantly with chronic gastrointestinal symptoms rather than typical urological manifestations, highlighting the importance of considering renal malignancy in patients with persistent unexplained abdominal symptoms and significant weight loss. Second, this case emphasizes the diagnostic value of thorough physical examination, as identification of a palpable flank mass redirected the diagnostic pathway toward renal imaging. Third, complete surgical resection resulted in resolution of gastrointestinal symptoms and substantial weight regain, supporting the role of tumor-related mass effect and visceral compression in the patient’s presentation. Finally, the unusual eosinophilic/chromophobe-like morphology highlights the importance of integrating histopathological findings with immunohistochemical evaluation to achieve accurate tumor classification. Overall, this case adds to the limited literature on giant ccRCC by documenting a very large tumor in a young adult with predominant gastrointestinal symptoms, no gross hematuria, no renal vein thrombus, negative margins, negative sampled lymph nodes, and early postoperative clinical improvement.

## Conclusion

Giant RCC is an uncommon presentation, particularly in young adults. This case describes a 31-year-old man with a massive ccRCC presenting predominantly with gastrointestinal symptoms and marked weight loss, without gross hematuria or venous tumor thrombus. Histopathological and immunohistochemical findings supported the diagnosis of ccRCC with focal eosinophilic/chromophobe-like morphology. Complete surgical excision resulted in resolution of gastrointestinal symptoms, significant weight regain, preserved renal function, and no evidence of recurrence during 6-month follow-up. This case highlights the importance of considering renal malignancy in patients with persistent nonspecific abdominal symptoms and a palpable mass, emphasizes the diagnostic value of careful clinical examination, and reinforces the need for accurate pathological classification and long-term oncological surveillance.

## Statement of Ethics

The Ethics Committee at Damascus University does not require ethical approval for reporting individual cases or case series. The CARE Checklist for this case report is available as online supplementary material (for all online suppl. material, see https://doi.org/10.1159/cro/abzag020). Written informed consent was obtained from the patient for publication of this case report and any accompanying images.

## Conflict of Interest Statement

The authors have no conflicts of interest to declare.

## Funding Sources

This study was not supported by any sponsor or funder.

## Author Contributions

Salem Hakouk contributed to the clinical evaluation of the patient, surgical management, acquisition of clinical and operative data, interpretation of clinical findings, and critical revision of the manuscript. Omar Al-Abed contributed to patient management, surgical care, collection of clinical data, interpretation of operative findings, and revision of the manuscript for important clinical content. Mohammad Alaa Aldakak contributed to data organization, literature review, drafting of the manuscript, preparation of the final manuscript, reference formatting, and correspondence with the journal. Anas Saad contributed to perioperative patient care, clinical data collection, follow-up assessment, and revision of the manuscript. Ahmad Awaid contributed to clinical data acquisition, collection and organization of radiological and operative images, literature review support, and manuscript editing. Lina Assad performed and interpreted the histopathological and immunohistochemical evaluation, selected the pathological images, and critically revised the pathology-related sections of the manuscript. Ibrahim Barghouth supervised the overall clinical management of the case, contributed to surgical decision-making, reviewed the manuscript for scientific and clinical accuracy, and provided final supervision and approval before submission. All authors read and approved the final version of the manuscript.

## Data Availability

All data generated or analyzed during this study are included in this article and its online supplementary material. Further inquiries can be directed to the corresponding author.
